# Sedimentary archaeal *amoA* gene abundance reflects historic nutrient level and salinity fluctuations in Qinghai Lake, Tibetan Plateau

**DOI:** 10.1038/srep18071

**Published:** 2015-12-15

**Authors:** Jian Yang, Hongchen Jiang, Hailiang Dong, Weiguo Hou, Gaoyuan Li, Geng Wu

**Affiliations:** 1 State Key Laboratory of Biogeology and Environmental Geology, China University of Geosciences, Wuhan, 430074, China; 2 State Key Laboratory of Biogeology and Environmental Geology, China University of Geosciences, Beijing, 100083, China; 3 Department of Geology and Environmental Earth Science, Miami University, Oxford, OH45056, USA

## Abstract

Integration of DNA derived from ancient phototrophs with their characteristic lipid biomarkers has been successfully employed to reconstruct paleoenvironmental conditions. However, it is poorly known that whether the DNA and lipids of microbial functional aerobes (such as ammonia-oxidizing archaea: AOA) can be used for reconstructing past environmental conditions. Here we identify and quantify the AOA *amoA* genes (encoding the alpha subunit of ammonia monooxygenases) preserved in a 5.8-m sediment core (spanning the last 18,500 years) from Qinghai Lake. Parallel analyses revealed that low *amoA* gene abundance corresponded to high total organic carbon (TOC) and salinity, while high *amoA* gene abundance corresponded to low TOC and salinity. In the Qinghai Lake region, TOC can serve as an indicator of paleo-productivity and paleo-precipitation, which is related to historic nutrient input and salinity. So our data suggest that temporal variation of AOA *amoA* gene abundance preserved in Qinghai Lake sediment may reflect the variations of nutrient level and salinity throughout the late Pleistocene and Holocene in the Qinghai Lake region.

Over the last decade, ancient DNA preserved in the sedimentary record has been extensively employed to reconstruct past biological communities, especially for non-fossilized organisms^1^,^2^,^3^,^4^ and to interpret the effects of paleo-environmental condition on marine/lacustrine planktonic communities^5^,^6^,^7^,^8^,^9^,^10^,^11^,^12^,^13^,^14^. Most of these previous ancient DNA-based studies have mainly focused on phototrophs (e.g. dinoflagellates, haptophytes, sulfur bacteria). Few studies have investigated ancient microbial communities and their response to paleo-environmental conditions from a functional gene perspective (such as ammonia-oxidizing archaea: AOA) in marine or lacustrine sediment records^15^,^16^.

AOA are well known for their capability of oxidizing NH_4_^+^ aerobically to NO_2_^−^, and phylogenetically they are classified into *Thaumarchaeota*, a novel phylum within the *Archaea* domain^17^,^18^. All known AOA strains carry the *amoA* gene encoding the alpha subunit of ammonia monooxygenase, which catalyses ammonia oxidation. The *amoA* gene is widely used as a molecular biomarker to study AOA distribution in various environments^19^,^20^. However, it is unknown whether all *amoA*-carrying thaumarchaeota have the capability of ammonia oxidation. For example, *Candidatus* Cenarchaeum symbiosum carries the *amoA* gene, but it does not oxidize ammonia^21^. Accordingly, the *amoA* gene-carrying archaea should be considered as *amoA*-encoding archaea (AEA)^22^.

Recent studies have shown that AEA might play a major role in ammonia oxidation in high-elevation lakes^23^,^24^,^25^ and their abundance and diversity are influenced by limnological conditions, such as salinity^26^,^27^, pH^23^, ammonia concentration^28^, temperature^24^,^29^, trophic status^30^,^31^,^32^, and water depth^33^,^34^. These observed correlations of AEA abundance and diversity with environmental factors suggest a possibility of using the AEA DNA preserved in the sedimentary record to reconstruct paleo-environmental change.

In addition to the *amoA* gene, AOA synthesize a unique lipid biomarker thaumarchaeol (previously called crenarchaeol), which is an isoprenoid glycerol dialkyl glycerol tetraether (iGDGT) that contains four cyclopentyl rings and one cyclohexyl ring^35^. Thaumarchaeol was considered as a characteristic biomarker of Thaumarchaeota^35^,^36^,^37^,^38^,^39^,^40^. Because thaumarchaeol can be preserved for millions of years in the sedimentary record, it can be used in junction with other iGDGTs as a proxy for past environmental conditions, such as salinity and water temperature^41^,^42^. To date, thaumarchaeol has been detected in lake sediments worldwide^43^ and many studies have shown that environmental factors control thaumarchaeol distribution in either modern or ancient lakes^29^,^41^,^42^,^43^.

Qinghai Lake (36°32’ −37°15’N, 99°36’−100°47’E) is a perennial lake (salinity 14.2 g/L, pH 9.1)^26^ located on the northeastern Qinghai-Tibetan Plateau (QTP). The lake has a surface area of 4260 km^2^ with a catchment area of 29,660 km^2^, and has an average water depth of 21 m^44^. Qinghai Lake is situated at a critical and sensitive junction of four climatic systems: the East Asian Monsoon, the India monsoon, the Siberian Winter Monsoon, and the Westerlies^45^. The annual temperature changes from −11°C in winter to 12 °C in summer^42^. The mean annual precipitation is about 373 mm and rainfall mainly occurs in June, July and August, which is significantly influenced by monsoonal variations^46^. The Qinghai Lake sediments archive abundant palaeoclimatic records, especially the Asian monsoon variation^46^,^47^,^48^,^49^. A recent study showed that thaumarchaeol was detected in the sediments of Qinghai Lake 12,000 years BP (before present) and was successfully used for reconstructing past lake level^42^. However, the lipid biomarker-based approach could not determine the diversity and abundance of AEA, although it could be an important indicator for paleo-environmental condition. Therefore, analyses of the thaumarchaeotal *amoA* gene preserved in Qinghai Lake sediments provide insights into the response of AEA community to paleo-environmental change.

The objective of this study was to investigate the AEA diversity and abundance in the sediments of Qinghai Lake and their response to paleo-climate and paleo-environmental changes. An integrated approach was employed including geochemistry and 16S rRNA gene/*amoA* gene-based analyses.

## Results

The calibrated ages obtained from this study are presented in Table 1. A regression line between ^14^C age and depth revealed a reservoir effect of 538 years, which was similar to those observed previously for this lake^47^,^48^,^50^. Our age-depth relationship was fit by using an exponential function (Supplementary Fig. S1). This age model was selected because it presents the best fit (R^2^ = 0.989) and gives an age of 10,300 cal. cal. kyr BP for the distinct dolomite layer (data not shown), consistent with previous studies^47^. Accordingly, this sediment core covered a time span of approximately 18,500 years and time resolution was approximately 32 years per centimeter of sediment.

**Figure 1:**
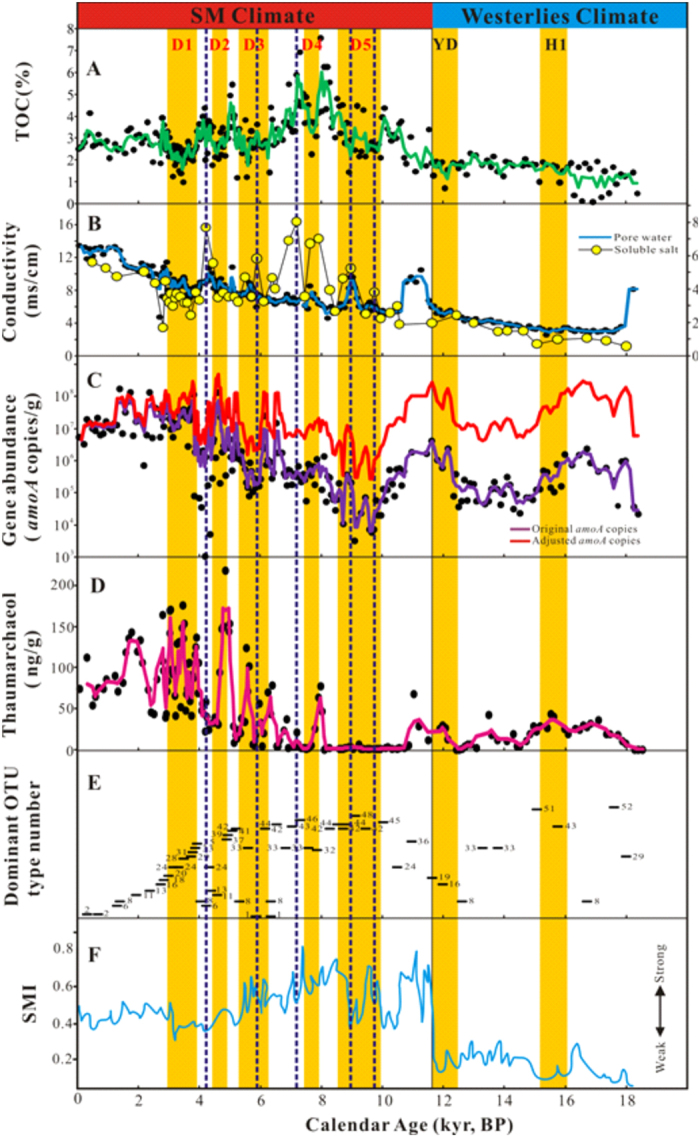
Geochemistry and microbial properties of the studied sediment core. (**A**) TOC; (**B**) Conductivity of the sediment pore water and soluble salts (Li *et al*., 2014, unpublished); (**C**) Original and adjusted *amoA* gene abundances as quantified by qPCR; (**D**) Thaumarchaeol contents^42^; (**D**) Distribution of dominant *amoA* OTU types; (**F**) Summer monsoon index (SMI) of the Qinghai Lake region^46^. All data points were plotted against calendar year ages, and solid lines in chart (**A**–**D**) were plotted with average values of every three conjoint points. YD: Younger Dryas; H1: Heinrich event1; D1-D5: dry periods^46^,^47^,^48^,^49^,^51^,^52^,^53^. Summer monsoon (SM) climate was dominant since 11.5 cal. kyr BP in Qinghai Lake region, while westerlies climate was dominant during 18.5−11.5 cal. kyr BP^46^.

The TOC contents of the sampled sediments ranged from 0.1% to 7.6% (Supplementary Table S1). The TOC content was the highest (7.6%) in the sample QHLS353 and was the lowest (0.1%) at the bottom of the core. High TOC values were observed during 10.5–4.0 cal. kyr BP (Fig. 1A) and corresponded to an overall warm Holocene period, which is consistent with previous studies^46^,^47^. Low TOC values were observed during 18.5–11.5 cal. kyr BP (Fig. 1A) and corresponded to many cold-dry events (e.g. Younger Dryas, Heinrich event, yellow columns in Fig. 1)^46^,^47^,^49^. Five periods (D1-D5, indicated as yellow columns in Fig. 1) with low TOC were identified in the last 11.5 cal. kyr BP, and they corresponded to well-known cold-dry climatic events described in previous studies^46^,^47^,^48^,^49^,^51^,^52^,^53^. In addition, pore water conductivity increased from the bottom to the top of the sediment core and ranged from 2.3 to 13.4 ms/cm (Fig. 1B and Supplementary Table S1).

Total thaumarchaeotal 16S rRNA gene abundance ranged from 3.41 × 10^6^ to 1.22 × 10^9^ copies per gram of sediment (Supplementary Table S1) with the highest value at the depth of 31 cm and lowest at the depth of 179 cm. The *amoA* gene abundance ranged 3.23 × 10^3^ to 1.73 × 10^8^ copies per gram of sediment (Supplementary Table S1) with the highest value at the depth of 205 cm and the lowest at the depth of 405 cm. Generally speaking, the thaumarchaeotal 16S rRNA and *amoA* gene abundances decreased with increased depth. Archaeal *amoA* gene abundance was significantly correlated (r = 0.360, *P* = 0.0001) with thaumarchaeotal 16S rRNA gene abundance (Supplementary Fig. S2).

**Figure 2:**
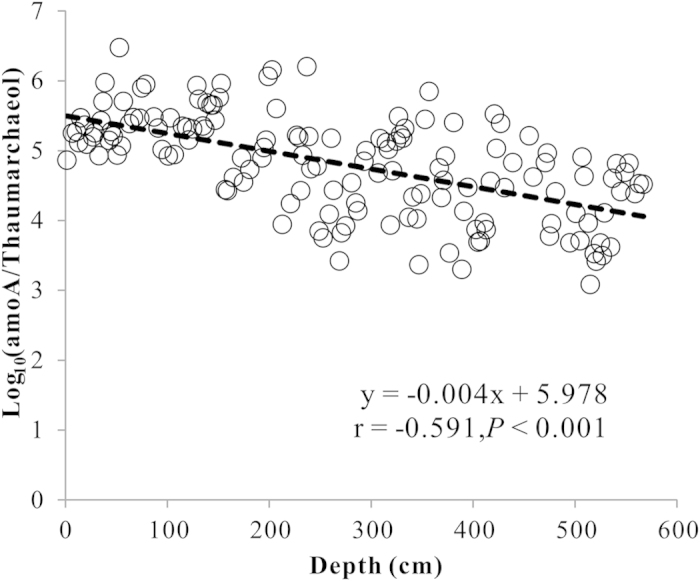
Linear correlation between depths and the ratios of original *amoA* gene abundance to thaumarchaeol concentration.

Biological macromolecules like DNA can be readily degraded due to hydrolysis and oxidation in ancient sediments, and the extent of DNA degradation generally increases with sediment age or depth^5^,^9^,^13^,^54^. Thus, the extent of sedimentary DNA degradation should be first assessed when gene abundance can be correlated with paleo-environmental conditions. Previous studies have shown that the ratio of specific lipid biomarker to their corresponding gene abundance can be used to assess DNA preservation in lacustrine and marine sediments^10^,^14^. For example, haptophyte ribosomal RNA genes and their specific lipid biomarker long-chain alkenones (LCAs) are commonly compared to evaluate the extent of sedimentary DNA degradation in marine or lacustrine sediments^10^,^14^, because LCAs can be preserved in ancient lacustrine sediments as old as the Miocene^55^. Similarly, AOA-specific lipid (thaumarchaeol) is a suitable lipid biomarker for assessing DNA degradation of *Thaumarchaeota*, because glycolipid of thaumarchaeol is resistant to degradation and can be preserved for much longer time than DNA in the sediment^41^. Accordingly, in this study the ratio of AEA *amoA* gene abundance to the published thaumarchaeol concentration^42^ obtained from the same sediment core was calculated to evaluate DNA preservation in the Qinghai Lake sediments. The results showed that the ratio decreased with depth along the sediment core (Fig. 2), suggesting that DNA underwent degradation in the Qinghai Lake sediments.

In order to make meaningful comparison of the *amoA* gene abundance throughout the core, the *amoA* gene abundance was adjusted on the basis of a linear correlation (r = −0.591, *P *< 0.0001) between the ratio of *amoA* gene abundance to the thaumarchaeol concentration^42^ and depth (Fig. 2). The adjusted *amoA* gene abundance ranged from 1.5 × 10^5^ to 9.79 × 10^8^ (Fig. 1C and Supplementary Table S1) and showed a negative correlation (r = −0.367, *P* = 0.0001) with TOC content. High or low *amoA* gene abundances were observed in seven well-known cold-dry periods (D1-D5, YD, and H1; yellow bars in Fig. 1), which were identified according to low TOC values and previous studies^46^,^47^,^48^,^49^,^51^,^52^,^53^. For examples, high AEA abundance corresponded to the D1, D2, YD and H1 periods, which had weak fluctuation of dissolved salt and pore water conductivity (Fig. 1B); whereas low AEA abundance corresponded to D3, D4 and D5 periods, which had strong fluctuation of dissolved salt and pore water conductivity (Fig. 1B). The above relationships were also evidenced by the thaumarchaeol profile (Fig. 1D).

**Figure 3:**
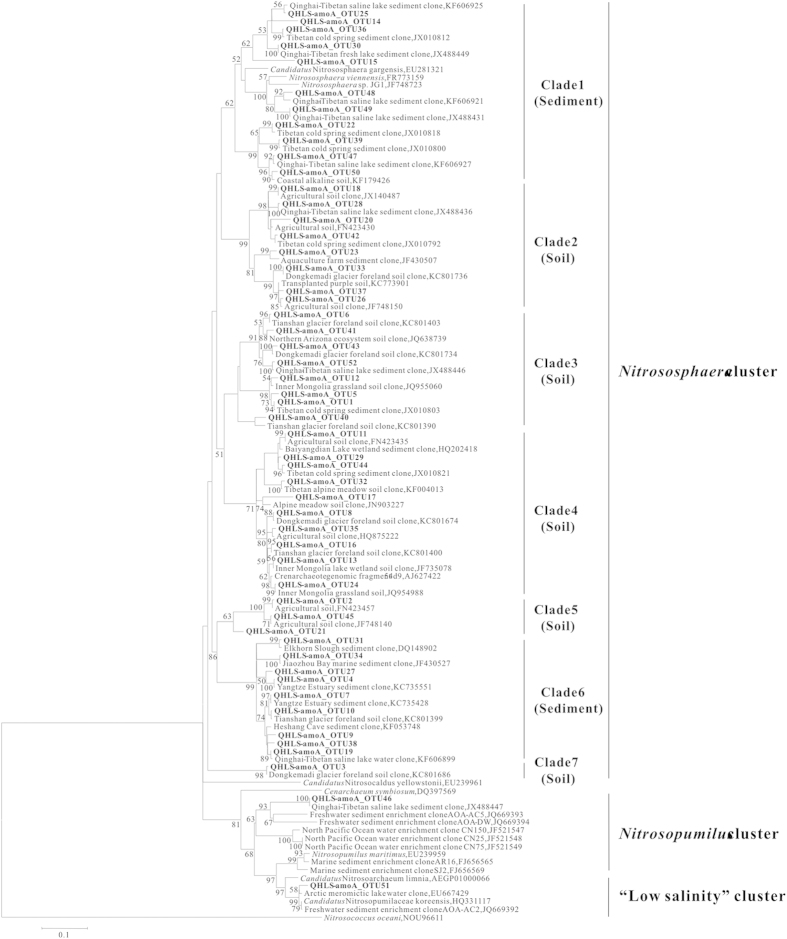
Maximum-likelihood tree showing the phylogenetic relationships of the *amoA* gene clone sequences obtained in this study to their closely related sequences from the GenBank database. One representative clone type within each OTU is shown, and the OTUs from this study are bolded. The scale bar indicates the Jukes-Cantor distances. Bootstrap values of (1000 replicates) >50% are shown. The bacterial *amoA* gene from *Nitrosococcus oceani* was used as outgroup.

A total of 1233 clones were randomly selected from libraries and were classified into 52 OTUs (Supplementary Table S2). The coverage values of the clone libraries were 87–100% (Supplementary Table S2). Calculated diversity indices varied among the samples and roughly decreased with sample depth (Supplementary Table S2). Phylogenetic analyses showed that the obtained *amoA* gene clone sequences were mostly grouped into the *Nitrososphaera* cluster (Clades 1–7), and a small fraction (<5% of total clone sequences) was grouped into the *Nitrosopumilus* cluster and a “low salinity” cluster^56^ (Fig. 3). Within the *Nitrososphaera* cluster, the *amoA* genes derived from this study in the Clades 1 and 6 were closely related (~98% nucleic acid identity) to those from other saline sediments, such as the sediments of the QTP saline lakes^26^ and oceans^57^. The sequences in Clades 2–5 and 7 were closely related (95–98%) to clone sequences from soils, such as glacier foreland soils (described in NCBI database) and agricultural soils^58^. In the *Nitrosopumilus* cluster, the obtained *amoA* gene sequences were closely related (~99%) to those from the QTP saline lake sediments^26^. In the “low-salinity” cluster, the *amoA* gene clone sequences were closely related (~98%) to those from the Arctic meromictic lake water^33^.

Distinct AEA community compositions were observed in the studied samples with different TOC contents (Supplementary Fig. S3). For examples, the low-TOC (<2%) samples were dominated with OTUs 19, 51 and 52; the medium-TOC (2–5%) samples were dominated with OTUs 8, 24 and 33; while the high-TOC (>5%) samples were only dominated with OTUs 41, 42 and 46.

## Discussion

The Qinghai Lake water column currently is oxic, but within a few centimeters below the water-sediment interface, the sediments become anoxic as determined from pore water chemistry^51^, consistent with a recent study which indicated that oxygen concentration was zero below one centimeter into lake sediments^59^. Furthermore, all known AEA require oxygen for growth, although previous studies indicated some AEA might prefer low oxygen environments^20^,^60^. So it is unlikely for AEA to survive in anoxic lake sediments. In this study, all of the selected samples were under 7 cm below sediment surface. Therefore, the retrieved *amoA* genes should have originated mainly from ancient AEA population but currently buried in the sediments.

The observed temporal variation of AEA abundance in this study might be caused by changes of paleo-environmental conditions, such as nutrient level and salinity. Nutrient level may be a dominant factor affecting the AEA growth over the past 18, 500 years in the Qinghai Lake. Nutrient level affects AEA population in modern ecosystems^32^. High AEA abundance is generally observed in oligotrophic open oceans, because AEA prefer a low level of nutrition^20^,^61^. Recent studies in lakes also indicated that AEA abundance was negatively correlated with nutrient level^30^,^31^,^32^. During 18.5–11.5 cal. kyr BP, the Westerlies played a major role in controlling climate in the Qinghai Lake region^46^, and weak ASM and low precipitation prevailed in this period^46^,^47^,^49^ and resulted in many intensified cold-dry events, such as Younger Dryas and Heinrich 1 (marked by the yellow bars labeled YD and H1 in Fig. 1)^46^,^47^,^48^,^49^,^51^,^52^. Therefore, nutrients were inferred to be poorly transported into Qinghai Lake by terrestrial runoff, and this oligtrophic condition would have favored the AEA growth because of their oligotrophic physiology^30^,^31^,^32^. Indeed, high *amoA* gene abundance (adjusted *amoA* genes almost >10^7^ copies/g) was observed in this period, which corresponds to low TOC values (almost* *< 2%) (Fig. 1A,C). Furthermore, A previous study indicated that ASM abruptly increased around 11.5 cal. kyr BP^46^, which might have brought ample rainfall to the Qinghai Lake region resulting in elevated nutrient level in the lake, and thus inhibited the growth of oligotrophic AEA^30^,^31^,^32^. Accordingly, it is reasonable to observe a negative correlation (r = −0.367, *P* = 0.0001) between the AEA abundance and TOC content (Fig. 1A,C) in this study.

Salinity fluctuation may be another factor influencing the AEA growth over the past 18, 500 years in the Qinghai Lake. Salinity shapes AEA population in modern environments (e.g. lakes, estuaries, oceans)^26^,^27^,^60^. However, the response pattern of AEA abundance to salinity may be different, even conflicting. For example, AEA *amoA* gene abundance was negatively correlated with salinity in the San Francisco Bay estuary^56^, while an opposite trend was observed in another six different estuaries^62^. Such discrepancy could be ascribed to the heterogeneity of those estuaries. However, affirmatory reasons for this inconsistency are difficult to obtain with limited environmental data provided in those studies. One of our previous study showed that AEA *amoA* gene abundance decreased with increasing salinity in the Qinghai-Tibetan lakes with different salinity^26^, which was comparable to the data obtained in this study in that the studied lakes (including Qinghai Lake) were located in one region and methodology was the same. So it is reasonable to observe negative correlation between salinity and AEA *amoA* gene abundance throughout the Qinghai Lake sediment core. A previous study suggested that the Westerlies may be a cold-air conveyer between the North Atlantic and the northeastern QTP in the glacial time^46^. Cold temperature might have predominated this period and weakened lake evaporation. Therefore, the resulting low salinity during 18.5–11.5 cal. kyr BP, as evidenced by low porewater conductivity (almost* *< 5 ms/cm) and soluble salt content (almost* *< 2 ms/cm) (Fig. 1B,C), might have favored AEA growth, because AEA prefer low salinity for growth^56^. Since 11.5 cal. kyr BP, strong evaporation events (indicated by high soluble salt content, Fig. 1B) can result in salinity increase^63^ and it would inhibit AEA growth and thus decrease AEA abundance (indicated as the vertical dash lines in Fig. 1).

The temporal changes of AEA abundance and population could reflect the temporal variations of salinity and nutrient levels in historical time of the Qinghai Lake. Previous studies have shown that the climate in the Qinghai Lake region is primarily controlled by Asian summer monsoon (ASM) during the Holocene^46^,^47^,^49^. TOC is a good indicator for monsoonal precipitation in the Qinghai Lake region through its linkage to nutrient input and stimulated primary productivity^46^,^64^. Strong ASM brings large amounts of precipitation, and thus enhances terrestrial input (e.g. organic matter, inorganic salts), which can bring abundant nutrients to the lake, promote primary productivity, and accelerate the growth of phototrophic bacteria and algae, especially during the time period of 10.5–4.2 cal. kyr BP^51^. Both terrestrial input and aquatic productivity would result in a high TOC in the lake sediments. Conversely, weak ASM would result in a low nutrient level and primary productivity, and thus low TOC. In addition, strong ASM was most likely associated with increased temperature in the Northern Hemisphere^46^ and thus enhances evaporation, which could be evidenced by high dissolved salt content^63^. The enhanced evaporation would increase lake salinity, which in turn could decrease the abundance of some salinity-sensitive microbes (e.g. AEA). Therefore, past climatic change may lead to fluctuations of nutrient level and salinity in the Qinghai Lake, where some specific microbial community (e.g. AEA) could respond to the variations of these paleo-limnological conditions.

It is noteworthy that the AEA abundance responded differently to different cold-dry events (shown as D1-D5 in Fig. 1) since 11.5 cal. kyr BP. Such contrasted response may result from distinct intensities of evaporation, which induced different patterns of salinity fluctuation. For examples, in the D1 and D2 periods, evaporation did not show abrupt increase, whereas evaporation in the D3-D5 periods experienced abrupt enhancements (Fig. 1B). Thus a conclusion can be drawn that the fluctuation of nutrient levels and salinity simultaneously influenced the AEA abundances in the Qinghai Lake since 18.5 cal. kyr BP.

In addition to AEA abundance, the AEA community structures in Qinghai Lake might also have been affected by nutrient level and salinity variations that resulted from climate change. For example, distinct OTU types predominated in the samples with different nutrient status (Fig. 1E). This observation is consistent with a previous study, which indicated that AEA community composition is distinct with different lake trophic status^32^. However, only a few OTU types repeatedly occurred in different layers with a similar trophic condition (e.g. high or low TOC) or in similar climatic conditions (e.g. cold-dry or warm-wet climates). The possible reason for this phenomena may be that other paleo-limnological conditions (e.g. salinity) affect the AEA community composition, but these conditions did not occurred coincidently with trophic condition or cold-dry climates of Qinghai Lake. For example, salinity was increasing since 18.5 cal. kyr BP in the Qinghai Lake, which indicated by increasing dissolved salt and pore water conductivity (Fig. 1B). Therefore, these heterogeneous distribution of AEA OTU types may be ascribed to salinity difference, which has been shown as an important factor shaping the AEA community structures in saline lakes^27^.

In conclusion, the variation of AEA abundance throughout the Qinghai Lake sediment core could correspond to the fluctuations of historic trophic status and salinity in Qinghai Lake: high trophic status, a result of high precipitation and strong ASM, inhibited the AEA growth (low abundance); whereas dry and cold climates led to oligotrophic conditions and thus favored the AEA growth (high abundance). Therefore, the AEA abundance generated from ancient DNA preserved in lake sediments may be a promising proxy for reflecting variations of paleo-climate and paleo-environmental conditions in lakes.

## Methods

In August 2011, a 580 cm long sediment core was recovered from the southeastern corner of Qinghai Lake (36°39.6’N, 100°36’E). The core was cut into 30–50 cm segments and their ends were sealed in the field using sterilized plastic lids. The core segments were immediately kept in the dark, stored on dry ice during transportation to laboratory, and stored at −80 °C until further analysis.

The sediment core segments were sub-sampled in a UV-sterilized bio-hood in a dedicated room. Prior to use, bench space was ethanol-sterilized, and the room was UV sterilized for 12 hours. Laboratory tools used for sub-sampling, such as lab clothes, mask, scalpel, spoon, and tubes, were autoclaved or UV-sterilized before use. The core segments were sliced into approximately 2-cm depth intervals. The outer layers of the sub-samples were discarded and the inner portions were collected for further analyses. The scalpels and spoons were sterilized with ethanol and flame between two consecutive samples to avoid cross contamination. The sub-samples were coded as follows with QHLS007 as an example: Qinghai Lake sediment from 7 to 9 cm in depth. A total of 290 subsamples were obtained from the core. All subsamples were stored at −80 °C in the laboratory until further analysis.

Six sub-samples (QHLS057, QHLS115, QHLS221, QHLS351, QHLS487, and QHLS575) were selected for ^14^C accelerator mass spectrometry (AMS) dating at Beta Analytic Radiocarbon Dating Laboratory (Miami, Florida, USA). Lake sediment age was calibrated to calendar years B.P. (before present defined as of 1950) by using the IntCal13 calibration curve on the Calib7.0.2 program^65^.

All subsamples (~2 cm interval) were selected for geochemical and molecular analyses. TOC analysis was performed using a 2400 Series II CHNS/O Analyzer (PerkinElmer, Waltham, MA, USA). Briefly, sediment samples were acidified with 1 N HCl to remove carbonates, rinsed three times with deionized water, and dried at 80 °C. Finally, the dried samples (~20 mg per sample) were sealed in tin foil and loaded onto the analyzer for analysis. Pore water samples were obtained by centrifugation (5000 g) of sediment sub-samples. After dilution 50 times with deionized water these samples were measured for conductivity with a DDS-307A Conductometer (Shanghai Precision & Scientific Instrument CO.LTD, China).

Stringent precautions^9^,^13^ were taken throughout the molecular experiments (e.g. DNA extraction and PCR reaction): a bio-hood with a laminar flow cabinet was first cleaned with ethanol and then UV-sterilized at least for 4 hours. All tools (such as pipettes, centrifuge, vertex, pipette tips, tubes and gloves etc.) used in this study were autoclaved or UV-sterilized for 4 hours before use. Genomic DNA was extracted from sediment subsamples (~0.5 g sediment per sample) with the use of Fast DNA SPIN Kit for Soil (MP Biomedicals, USA) according to the manufacturer’s protocols. Two DNA extractions were carried out for blank controls (without any sediments) to check for any contamination from reagents and glassware^5^.

The thaumarchaeotal 16S rRNA gene and *amoA* gene abundances were quantified for sediment sub-samples by qPCR with the use of primer sets of 771F/957R^66^ and Arch-amoAF/Arch-amoAR^57^, respectively. qPCRs were performed in a reaction volume of 20 μL, containing 10 μL of 2 × SYBR^®^ Premix Ex TaqTM (Takara, Japan), 0.4 μM of each primer, 0.4 μL of ROX Reference Dye II (50 × ), and 1 μL of DNA template. qPCRs were performed in duplicate on an ABI 7500 real-time PCR system (Applied Biosystems, Carlsbad, CA, USA). The qPCR was conducted by using previous conditions^26^,^66^. Standard curves were created by using serial dilutions (10^1^ to 10^7^ copies) of plasmids (pGEM-T) containing cloned crenarchaeotal 16S rRNA gene and *amoA* genes with correlation coefficients of R^2^ > 0.99. PCR efficiencies were 90–100%. The quality and length of the qPCR products were checked by dissociation curve analysis and 1% agarose gel electrophoresis. The PCR products of DNA extraction controls did not show any bands on agarose gels. The qPCR results were expressed as the number of (partial) gene copies per gram (copies g^−1^) of sediments.

The thaumarchaeotal *amoA* gene was amplified from the extracted DNA samples with the primer set of Arch-amoAF and Arch-amoAR^57^. All PCR reactions for the *amoA* genes were performed using the previously described conditions^26^. PCR products (635 bp) were examined using gel electrophoresis in 1% agarose and appropriate bands were excised. PCR gels were purified with Agarose Gel DNA purification Kit (TaKaRa, Japan). Fifty-six clone libraries (Supplementary Table S2) for the *amoA* gene were constructed according to previously published procedures^26^. Clones were randomly selected and screened for inserts by performing another round of PCR using the above primers. The positive PCR products were digested with restriction endonucleases *Taq I* (TaKaRa, Dalian, China) according to the manufacturer’s protocols, and then incubated at 65 °C for 60 min for restricted fragment length polymorphism (RFLP) analysis. The digests were analyzed by electrophoresis through a 2% (w/v) agarose gel. Unique RFLP patterns were identified visually. Subsequently, the rarefaction curves were constructed using clone numbers and E-values determined by the aRarefactWin (www.uga.edu/strata/software/Software.html) according to RFLP patterns (Supplementary Fig S4). The RFLP analysis was stopped when the rarefaction curves were (or almost) saturated. One representative clones of each RFLP pattern were selected for sequencing. The *amoA* gene inserts were sequenced with an ABI 3100 automated sequencer using the primer M13F: (5′-GTAAAACGACGGCCAG-3′).

All the obtained nucleotide sequences were checked and trimmed manually by using the BioEdit program (http://www.mbio.ncsu.edu/bioedit/bioedit.html). The trimmed sequences were used to perform BLAST (www.ncbi.nlm.nih.gov/blast/) against available *amoA* genes in the GenBank. Meanwhile, their closest references were retrieved for constructing phylogenic trees. The operational taxonomic units (OTUs) of *amoA* gene sequences were determined by using the software program DOTUR (nearest neighbor algorithm)^67^ with a cutoff value of 98%. One representative *amoA* sequence from each OTU was chosen and aligned with their references by using Clustal W implemented in the Bioedit program. Maximum-likelihood tree were constructed from *amoA* genes and their references by using the MEGA 6^68^. The clone sequences determined in this study were deposited in the GenBank database under accession numbers KJ191124-KJ191175.

Library coverage was calculated using the equation C = 1 − (n1/N), where n1 represents the number of OTUs that occurred only once in the clone library and N indicates the total number of clones analyzed in the library^69^. Diversity indices (Shannon, Simpson, Buzas and Gibson’s Evenness and Chao 1) were calculated by the PAST software package (http://folk.uio.no/ohammer/past/). Shannon index was calculated using the following equation: 
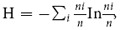
, where n_i_ is the number of clones within a given OTU and n is the total number of clones. Simpson index was calculated using 1 − D, where 
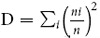
. Buzas and Gibson’s evenness is expressed as e^H^/S, where H is Shannon index and S is total number of OTUs. Chao1 is an estimate of total species richness, and it was calculated using the equation: Chao 1 = S + F_1_(F_1_ − 1)/(2 (F_2_ + 1)), where F_1_ is the OTU number of singleton sequences and F_2_ is the OTU number of doubleton sequences.

## Additional Information

**How to cite this article**: Yang, J. *et al*. Sedimentary archaeal *amoA* gene abundance reflects historic nutrient level and salinity fluctuations in Qinghai Lake, Tibetan Plateau. *Sci. Rep*. **5**, 18071; doi: 10.1038/srep18071 (2015).

## Supplementary Material

Supplementary Information

## Figures and Tables

**Table 1:** ^14^C AMS ages analyzed on TOC and calibrated ages for Qinghai Lake.

Depth (cm)	Conventional ^14^C age/yr, BP (1σ)	Reservoir-corrected ^14^C age by 538 yr, BP	Calendar age/cal yr, BP (2σ)	Median ages/cal yr, BP
58	3020 ± 30	2482	2453–2719	2586
116	4050 ± 30	3512	3699–3866	3783
222	4540 ± 30	4002	4418–4526	4472
352	7300 ± 40	6762	7571–7674	7623
488	12530 ± 50	11992	13726–13995	13861
576	15770 ± 80	15232	18454–18685	18570
