# Supplementary material for: Sedimentary archaeal amoA gene abundance reflects historic nutrient level and salinity fluctuations in Qinghai Lake, Tibetan Plateau
Source: Sci Rep. 2015 Dec 15;5:18071. doi: 10.1038/srep18071 (PMC4678299; doi:10.1038/srep18071)
Supplement: Supplementary Information [file srep18071-s1.pdf]

Sedimentary archaeal *amoA* gene abundance reflects historic nutrient level and salinity fluctuations in Qinghai Lake, Tibetan Plateau

Running title: AEA amoA gene reflects paleo-environmental conditions

Jian Yang[1], Hongchen Jiang[1*], Hailiang Dong[2,3*], Weiguo Hou[2], Gaoyuan Li[2], and Geng Wu[1]

[1]*State Key Laboratory of Biogeology and Environmental Geology, China University of Geosciences, Wuhan, 430074, China*
[2]*State Key Laboratory of Biogeology and Environmental Geology, China University of Geosciences, Beijing, 100083, China*
[3]*Department of Geology and Environmental Earth Science, Miami University, Oxford, OH45056, USA*

*Corresponding authors:

Hongchen Jiang: jiangh@cug.edu.cn; Tel: 86-27-67883061

Hailiang Dong: dongh@cugb.edu.cn; dongh@miamioh.edu

Author contributions

J.Y., H.J. and H.D. conceived and designed the experiments; J.Y., W.H., G.L. and G.W. analyzed the data. All of the authors assisted in writing the manuscript, discussed the results and commented on the manuscript.

Additional information

Competing financial interests: The authors declare no competing financial interests.

Caption for Supplementary Figures

Fig. S1 Age **-** depth model of the studied core.

Fig.S2 Linear correlation between thaumarchaeotal 16S rRNA and archaeal *amoA* gene abundance in the Qinghai Lake core.

Fig. S3 Distribution of dominant OTU types plotted against TOC content.

Fig. S4 Rarefaction curves of *amoA* gene libraries derived from this study.

Fig. S1 Age **-** depth model of the studied core.

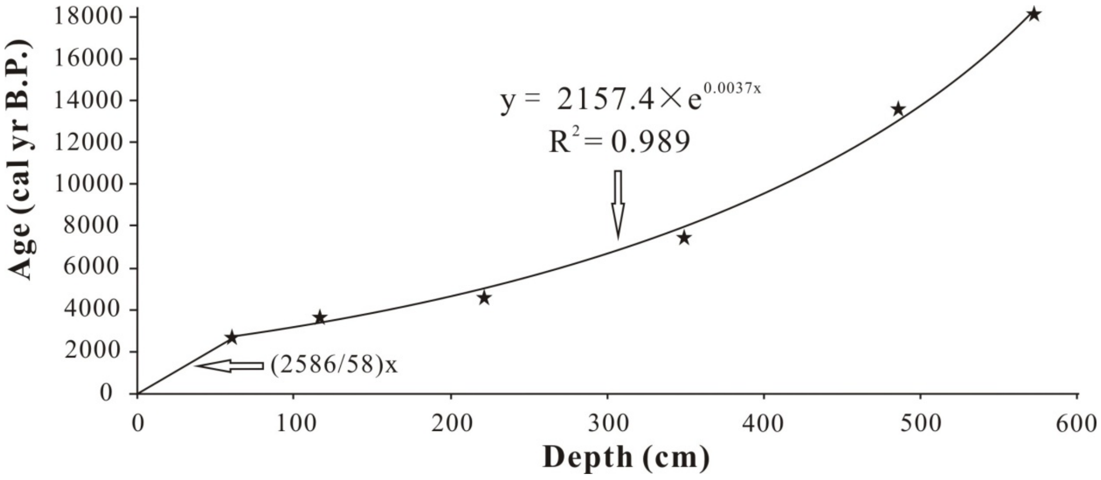

Fig.S2 Linear correlation between thaumarchaeotal 16S rRNA and archaeal *amoA* gene abundance in the Qinghai Lake core.

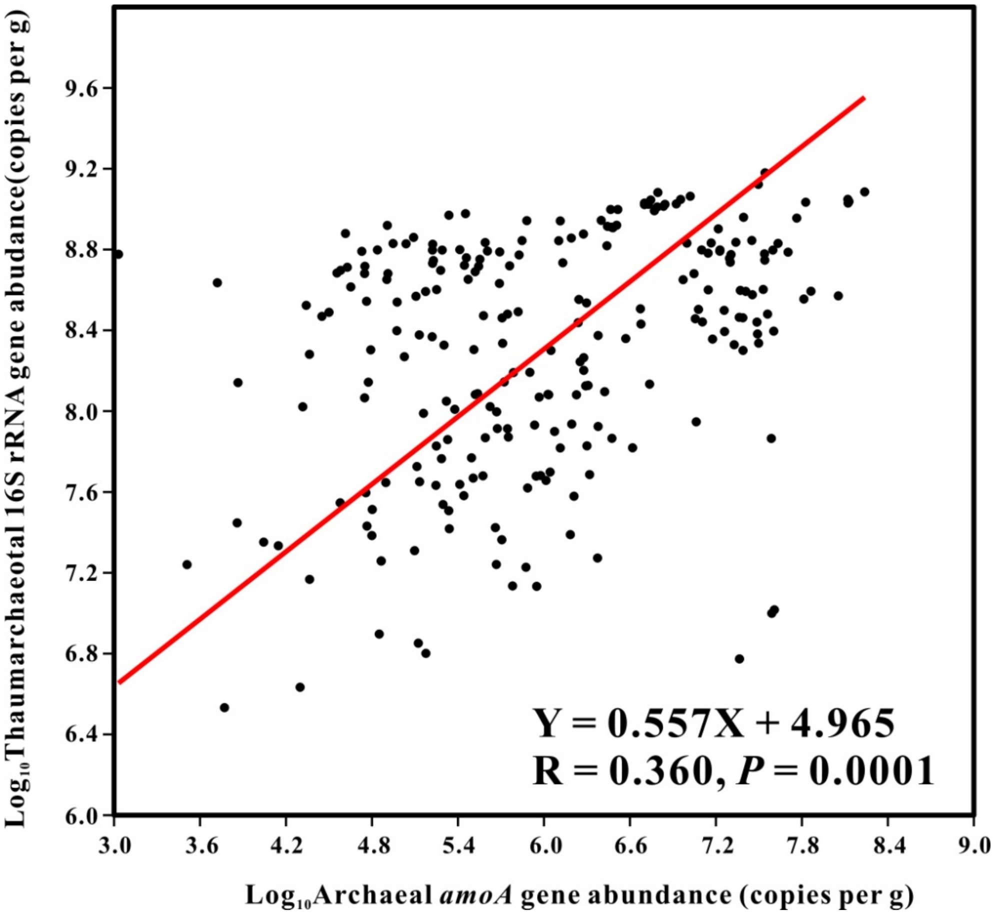

Fig. S3 Distribution of dominant OTU types plotted against TOC content.

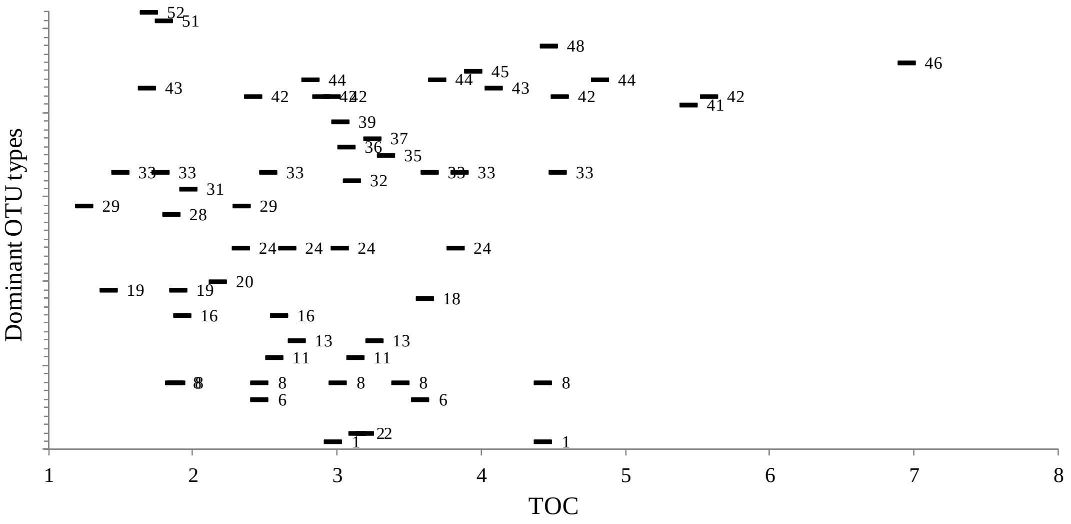

Fig. S4 Rarefaction curves of *amoA* gene libraries derived from this study.

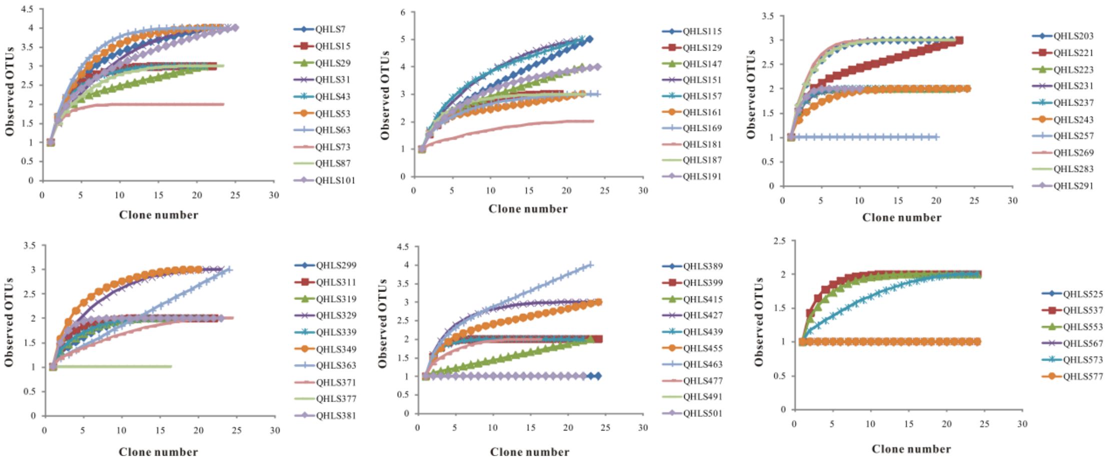

Table S1 Age, TOC contents, Pore water conductivities, Crenarchaeal 16S rRNA gene, raw amoA and adjusted amoA gene abundances of the sediment subsamples in the studied Qinghai Lake core.

Sample IDs	Kal. Age (Year BP)	Conductivity (ms/cm)	TOC(%)	*amoA* gene abundance (copies per g sediment)	SD	Adjusted *amoA* gene abundance (copies per g sediment)	Thaumarchaeotal 16S rRNA gene abundance (copies per g sediment)	SD (copies per g sediment)
QHLS001	44.6	13.5	2.5	5.36E+06	4.53E+04	5.41E+06	1.06E+09	1.91E+07
QHLS003	133.8	nm	2.6	no dna	no dna	no dna	no dna	no dna
QHLS005	222.9	13.3	2.8	3.27E+06	4.87E+04	3.43E+06	9.96E+08	8.82E+06
QHLS007	312.1	11.8	3.1	1.97E+07	2.09E+06	2.10E+07	5.71E+08	4.05E+07
QHLS009	401.3	13.1	4.2	1.47E+07	4.84E+05	1.59E+07	6.80E+08	1.23E+08
QHLS011	490.4	12.2	2.6	9.94E+06	1.19E+05	1.10E+07	6.79E+08	2.56E+07
QHLS013	579.6	13.1	2.7	8.37E+06	5.91E+05	9.44E+06	1.06E+09	4.95E+07
QHLS015	668.8	13.0	3.2	2.18E+07	1.53E+06	2.50E+07	6.85E+08	8.47E+07
QHLS017	758.0	13.3	2.8	6.17E+06	2.01E+05	7.21E+06	1.02E+09	1.85E+06
QHLS019	847.1	12.4	2.8	6.98E+06	9.73E+04	8.32E+06	1.06E+09	2.77E+07
QHLS021	936.3	nm	2.8	1.69E+07	2.41E+05	2.05E+07	6.19E+08	6.10E+07
QHLS023	1025.5	13.2	2.1	1.05E+07	4.56E+05	1.30E+07	1.16E+09	9.93E+06
QHLS025	1114.7	nm	nm	8.97E+06	1.96E+06	1.13E+07	1.12E+09	4.55E+07
QHLS027	1203.8	13.4	2.7	1.40E+07	2.19E+06	1.79E+07	6.05E+08	3.77E+07
QHLS029	1293.0	13.0	2.5	1.26E+07	1.17E+06	1.64E+07	6.28E+08	3.17E+07
QHLS031	1382.2	12.9	2.5	1.73E+08	5.03E+06	2.30E+08	1.22E+09	1.13E+08
QHLS033	1471.3	11.7	nm	6.82E+06	1.68E+05	9.24E+06	1.03E+09	7.25E+06
QHLS035	1560.5	11.1	2.3	3.13E+07	6.04E+05	4.32E+07	1.33E+09	3.75E+07
QHLS037	1649.7	10.9	3.4	6.70E+07	3.68E+06	9.42E+07	1.08E+09	5.18E+07
QHLS039	1738.9	10.8	3.5	1.32E+08	2.44E+06	1.89E+08	1.12E+09	3.38E+07
QHLS041	1828.0	10.7	2.4	5.89E+06	6.10E+06	8.59E+06	9.81E+08	6.00E+08
QHLS043	1917.2	10.7	3.1	1.64E+07	1.04E+06	2.44E+07	7.98E+08	1.74E+07
QHLS045	2006.4	10.3	3.2	2.47E+07	7.06E+05	3.74E+07	9.11E+08	3.94E+07
QHLS047	2095.6	11.3	2.8	1.69E+07	2.42E+05	2.60E+07	6.28E+08	9.37E+06

QHLS049	2184.7	11.1	3.0	7.02E+05	4.68E+05	1.10E+06	6.99E+08	2.94E+08
QHLS051	2273.9	11.1	3.7	6.23E+06	3.35E+04	9.97E+06	1.21E+09	7.52E+06
QHLS053	2363.1	9.7	2.7	1.34E+08	1.67E+06	2.19E+08	1.09E+09	4.19E+07
QHLS055	2452.2	11.0	1.9	5.02E+06	4.08E+05	8.33E+06	1.05E+09	1.78E+07
QHLS057	2541.4	10.0	3.2	4.30E+07	2.83E+06	7.26E+07	6.77E+08	5.42E+06
QHLS059	2683.7	4.7	3.1	no dna	no dna	no dna	no dna	no dna
QHLS061	2703.7	9.8	1.9	5.55E+06	3.86E+05	9.74E+06	1.11E+09	2.81E+07
QHLS063	2723.7	9.9	2.6	3.95E+07	8.70E+05	7.06E+07	6.27E+08	4.14E+07
QHLS065	2744.0	10.0	nm	no dna	no dna	no dna	no dna	no dna
QHLS067	2764.3	9.8	2.8	2.82E+07	4.26E+05	5.23E+07	7.00E+08	1.33E+07
QHLS069	2784.9	7.3	3.9	1.99E+07	4.47E+05	3.77E+07	5.46E+08	1.16E+08
QHLS071	2805.6	10.3	nm	no dna	no dna	no dna	no dna	no dna
QHLS073	2826.4	10.8	3.6	1.14E+07	2.03E+06	2.23E+07	2.87E+08	5.21E+07
QHLS075	2847.4	9.8	2.9	3.39E+07	1.64E+06	6.76E+07	4.00E+08	7.81E+07
QHLS077	2868.5	10.2	nm	no dna	no dna	no dna	no dna	no dna
QHLS079	2889.8	9.8	2.4	7.28E+07	3.85E+06	1.51E+08	3.93E+08	6.74E+07
QHLS081	2911.3	9.7	nm	no dna	no dna	no dna	no dna	no dna
QHLS083	2932.9	11.5	3.3	3.45E+07	2.08E+06	7.42E+07	6.00E+08	1.76E+08
QHLS085	2954.7	nm	nm	no dna	no dna	no dna	no dna	no dna
QHLS087	2976.7	8.1	2.2	5.04E+07	1.13E+07	1.12E+08	6.13E+08	1.35E+07
QHLS089	2998.8	10.0	3.0	no dna	no dna	no dna	no dna	no dna
QHLS091	3021.0	9.0	2.3	2.02E+07	8.97E+04	4.67E+07	5.96E+08	9.61E+06
QHLS093	3043.5	9.2	nm	no dna	no dna	no dna	no dna	no dna
QHLS095	3066.1	8.9	2.3	9.34E+06	6.51E+05	2.24E+07	4.47E+08	7.28E+07
QHLS097	3088.9	7.2	2.5	1.81E+07	1.74E+06	4.42E+07	3.16E+08	1.50E+08
QHLS099	3111.8	9.3	1.3	no dna	no dna	no dna	no dna	no dna
QHLS101	3134.9	8.7	2.6	5.45E+06	2.63E+04	1.38E+07	1.36E+08	1.47E+07
QHLS103	3158.2	9.4	2.4	1.20E+07	5.32E+05	3.09E+07	3.19E+08	1.06E+08
QHLS105	3181.7	8.9	1.8	no dna	no dna	no dna	no dna	no dna
QHLS107	3205.3	8.8	2.4	1.11E+07	2.46E+06	2.98E+07	4.79E+08	2.72E+07

QHLS109	3229.1	8.3	1.9	no dna	no dna	no dna	no dna	no dna
QHLS111	3253.1	8.1	2.6	1.40E+07	1.57E+06	3.89E+07	3.99E+08	4.43E+07
QHLS113	3277.2	8.8	2.1	no dna	no dna	no dna	no dna	no dna
QHLS115	3301.6	8.4	2.3	1.15E+07	5.11E+05	3.32E+07	8.86E+07	6.01E+07
QHLS117	3326.1	8.7	1.5	no dna	no dna	no dna	no dna	no dna
QHLS119	3350.8	8.4	2.6	3.15E+07	1.48E+07	9.42E+07	2.17E+08	2.69E+07
QHLS121	3375.7	9.0	2.3	2.45E+07	2.13E+06	7.46E+07	2.00E+08	5.77E+06
QHLS123	3400.8	9.2	1.4	no dna	no dna	no dna	no dna	no dna
QHLS125	3426.0	8.5	2.3	3.09E+07	1.28E+07	9.78E+07	2.41E+08	2.21E+07
QHLS127	3451.5	9.4	1.0	no dna	no dna	no dna	no dna	no dna
QHLS129	3477.1	9.6	1.8	4.01E+07	3.81E+06	1.32E+08	2.49E+08	1.18E+07
QHLS131	3502.9	9.2	2.5	3.63E+07	7.55E+05	1.21E+08	3.02E+08	5.98E+07
QHLS133	3529.0	8.7	nm	no dna	no dna	no dna	no dna	no dna
QHLS135	3555.2	8.4	2.1	2.31E+07	3.09E+06	8.01E+07	5.94E+06	2.06E+07
QHLS137	3581.6	7.4	2.3	2.12E+07	1.11E+06	7.50E+07	2.13E+08	2.73E+07
QHLS139	3608.2	8.2	2.8	2.84E+07	6.02E+06	1.02E+08	3.77E+08	3.90E+07
QHLS141	3635.0	8.2	2.7	2.75E+06	4.89E+05	1.01E+07	6.59E+08	5.39E+07
QHLS143	3662.0	7.7	2.5	1.82E+07	2.68E+06	6.79E+07	2.48E+08	6.08E+07
QHLS145	3689.2	7.1	2.1	4.05E+07	2.08E+06	1.54E+08	1.04E+07	3.96E+07
QHLS147	3716.6	7.7	2.3	2.34E+07	1.83E+06	9.07E+07	3.96E+08	1.00E+07
QHLS149	3744.2	nm	2.1	3.88E+07	9.76E+06	1.53E+08	9.98E+06	3.94E+07
QHLS151	3772.0	7.8	2.0	6.50E+07	3.30E+06	2.61E+08	3.59E+08	8.06E+07
QHLS153	3800.0	8.3	2.3	1.13E+08	1.92E+07	4.63E+08	3.72E+08	6.95E+07
QHLS155	3828.2	8.1	2.4	8.06E+04	1.94E+04	3.36E+05	8.31E+08	5.01E+07
QHLS157	3856.7	7.8	2.5	1.89E+06	8.52E+05	8.04E+06	1.84E+08	1.25E+07
QHLS159	3885.3	7.4	2.6	2.77E+06	4.06E+05	1.20E+07	8.21E+08	9.41E+06
QHLS161	3914.2	8.4	3.3	1.27E+07	1.67E+06	5.61E+07	2.77E+08	3.99E+07
QHLS163	3943.2	8.0	nm	no dna	no dna	no dna	no dna	no dna
QHLS165	3972.5	8.0	3.6	1.95E+06	7.30E+05	8.93E+06	1.33E+08	5.45E+07
QHLS167	4002.0	8.4	3.0	6.87E+04	1.47E+04	3.20E+05	6.28E+08	1.93E+07

QHLS169	4031.8	9.0	3.0	1.98E+06	5.66E+05	9.38E+06	3.43E+08	1.24E+07
QHLS171	4061.7	9.0	nm	3.59E+04	1.40E+04	1.74E+05	4.83E+08	3.43E+07
QHLS173	4091.9	8.9	3.2	1.75E+06	5.10E+05	8.62E+06	3.57E+08	2.50E+07
QHLS175	4122.3	9.0	4.0	2.03E+06	2.17E+04	1.02E+07	1.34E+08	4.56E+07
QHLS177	4152.9	9.1	4.4	no dna	no dna	no dna	no dna	no dna
QHLS179	4183.7	8.9	2.0	1.07E+03	8.28E+02	5.58E+03	5.98E+08	8.58E+06
QHLS181	4214.8	9.1	3.6	1.30E+06	2.98E+05	6.87E+06	6.58E+07	1.59E+06
QHLS183	4246.1	nm	2.3	5.02E+06	9.75E+05	2.71E+07	1.07E+09	5.38E+07
QHLS185	4277.6	9.4	3.0	1.67E+05	3.27E+04	9.18E+05	5.39E+08	6.45E+06
QHLS187	4309.4	10.7	3.8	3.06E+07	1.47E+07	1.71E+08	2.76E+08	4.26E+07
QHLS189	4341.4	9.2	3.6	5.26E+03	6.62E+03	3.00E+04	4.32E+08	2.93E+07
QHLS191	4373.7	9.5	3.3	3.03E+06	3.09E+05	1.76E+07	8.09E+08	5.85E+06
QHLS193	4406.2	10.1	1.8	2.87E+05	6.70E+04	1.70E+06	5.74E+08	1.99E+07
QHLS195	4438.9	9.4	3.5	3.72E+06	6.67E+05	2.24E+07	2.29E+08	5.49E+07
QHLS197	4471.9	8.3	2.7	4.71E+06	5.22E+05	2.89E+07	3.21E+08	9.50E+07
QHLS199	4505.1	8.7	2.1	3.47E+07	2.60E+06	2.17E+08	5.59E+08	1.37E+08
QHLS201	4538.5	8.3	nm	1.27E+06	9.32E+05	8.06E+06	6.98E+08	1.96E+08
QHLS203	4572.2	7.8	2.6	1.32E+08	5.87E+07	8.58E+08	1.07E+09	2.60E+08
QHLS205	4606.2	8.5	2.0	3.49E+07	4.72E+06	2.30E+08	1.51E+09	1.96E+07
QHLS207	4640.4	9.2	2.7	5.80E+07	6.72E+06	3.90E+08	9.03E+08	1.47E+08
QHLS209	4674.9	8.6	3.3	1.95E+05	2.72E+04	1.33E+06	6.27E+08	4.61E+07
QHLS211	4709.6	9.4	2.3	2.30E+07	1.17E+06	1.61E+08	2.91E+08	6.60E+07
QHLS213	4744.6	8.3	3.5	1.88E+06	1.94E+05	1.34E+07	7.53E+08	2.38E+07
QHLS215	4779.8	8.4	2.6	2.96E+06	5.26E+04	2.14E+07	8.14E+08	4.63E+07
QHLS217	4815.3	8.7	3.0	2.98E+06	5.23E+05	2.20E+07	7.34E+07	2.02E+07
QHLS219	4851.1	8.2	2.2	3.50E+05	1.09E+04	2.63E+06	5.21E+08	3.50E+07
QHLS221	4887.1	8.1	3.2	2.65E+06	3.58E+05	2.03E+07	1.25E+08	5.77E+07
QHLS223	4923.4	8.1	3.0	2.56E+07	2.15E+06	1.99E+08	3.92E+08	1.19E+07
QHLS225	4960.0	6.9	nm	2.51E+06	1.68E+05	1.99E+07	8.80E+08	9.75E+06
QHLS227	4996.8	8.2	5.0	4.75E+06	9.57E+05	3.84E+07	2.70E+08	1.60E+07

QHLS229	5033.9	nm	3.5	1.30E+06	1.23E+05	1.07E+07	8.75E+08	2.84E+07
QHLS231	5071.3	10.9	5.4	4.89E+05	1.57E+03	4.10E+06	4.28E+08	7.88E+07
QHLS233	5109.0	8.3	3.9	1.18E+06	5.77E+05	1.01E+07	7.94E+07	4.35E+06
QHLS235	5146.9	7.8	4.0	1.56E+06	5.08E+05	1.36E+07	8.63E+07	1.30E+06
QHLS237	5185.2	7.3	3.0	3.85E+07	5.23E+06	3.42E+08	7.33E+07	1.93E+06
QHLS239	5223.7	7.6	1.2	3.21E+06	1.32E+05	2.90E+07	8.33E+08	7.33E+06
QHLS241	5262.5	7.5	3.4	2.08E+06	7.73E+04	1.92E+07	4.86E+07	1.06E+07
QHLS243	5301.6	7.6	3.4	1.10E+06	5.68E+05	1.03E+07	5.00E+07	2.14E+06
QHLS245	5341.0	7.4	nm	1.55E+06	8.86E+04	1.48E+07	7.19E+08	3.46E+07
QHLS247	5380.6	7.4	2.6	1.99E+06	5.30E+05	1.94E+07	6.74E+07	1.60E+06
QHLS249	5420.6	7.5	2.3	8.59E+05	3.95E+05	8.51E+06	8.54E+07	1.52E+07
QHLS251	5460.8	7.7	nm	no dna	no dna	no dna	no dna	no dna
QHLS253	5501.4	7.5	3.8	5.63E+05	1.60E+05	5.79E+06	7.45E+07	4.58E+06
QHLS255	5542.3	7.5	1.2	2.13E+05	3.10E+04	2.23E+06	7.23E+07	2.82E+07
QHLS257	5583.4	7.7	1.9	1.50E+05	2.30E+07	1.60E+06	6.33E+06	
QHLS259	5624.9	8.9	2.1	8.15E+04	4.31E+04	8.85E+05	4.80E+08	5.62E+06
QHLS261	5666.7	8.6	2.9	8.82E+05	1.07E+05	9.76E+06	4.77E+07	5.42E+06
QHLS263	5708.8	8.5	3.3	4.73E+05	6.91E+04	5.33E+06	8.20E+07	9.74E+06
QHLS265	5751.2	8.2	2.5	1.09E+05	1.12E+04	1.25E+06	6.73E+08	1.21E+07
QHLS267	5793.9	7.6	2.6	2.83E+05	2.82E+04	3.31E+06	9.50E+08	1.96E+07
QHLS269	5836.9	7.1	3.0	1.44E+05	6.28E+03	1.72E+06	9.76E+07	3.14E+07
QHLS271	5880.3	6.0	3.6	2.18E+05	5.19E+04	2.65E+06	2.62E+07	2.25E+06
QHLS273	5924.0	7.5	nm	no dna	no dna	no dna	no dna	no dna
QHLS275	5968.0	6.9	2.8	1.67E+05	5.51E+04	2.10E+06	6.27E+08	7.97E+06
QHLS277	6012.3	7.3	2.6	2.75E+05	1.04E+05	3.53E+06	3.82E+07	1.53E+06
QHLS279	6056.9	6.9	2.6	2.92E+06	9.41E+03	3.81E+07	9.96E+08	2.48E+07
QHLS281	6101.9	6.5	2.6	2.38E+06	9.41E+05	3.17E+07	8.40E+07	1.87E+07
QHLS283	6147.2	6.6	2.9	2.44E+07	6.57E+06	3.31E+08	2.90E+08	4.71E+07
QHLS285	6192.9	6.6	nm	7.59E+05	9.54E+04	1.05E+07	8.77E+08	4.92E+04
QHLS287	6238.9	7.1	2.7	1.06E+06	1.89E+05	1.50E+07	1.21E+08	5.47E+07

QHLS289	6285.2	nm	1.4	no dna	no dna	no dna	no dna	no dna
QHLS291	6331.9	nm	4.4	9.25E+05	3.00E+04	1.35E+07	1.17E+08	5.34E+07
QHLS293	6379.0	6.6	3.9	2.78E+05	9.51E+04	4.14E+06	5.27E+08	2.41E+07
QHLS295	6426.3	6.8	2.7	4.20E+05	5.63E+04	6.36E+06	1.05E+08	1.10E+07
QHLS297	6474.1	6.3	nm	no dna	no dna	no dna	no dna	no dna
QHLS299	6522.1	6.4	2.8	1.50E+07	2.96E+06	2.35E+08	2.27E+08	1.47E+07
QHLS301	6570.6	6.7	3.4	1.79E+06	3.37E+05	2.86E+07	1.76E+08	1.47E+06
QHLS303	6619.4	6.7	nm	no dna	no dna	no dna	no dna	no dna
QHLS305	6668.6	nm	nm	no dna	no dna	no dna	no dna	no dna
QHLS307	6718.1	6.7	2.9	1.73E+06	6.71E+05	2.93E+07	2.74E+08	1.61E+07
QHLS309	6768.0	6.7	3.7	4.03E+05	1.45E+04	6.94E+06	6.20E+08	1.66E+07
QHLS311	6818.3	7.0	3.6	3.24E+05	1.60E+03	5.68E+06	2.02E+08	4.10E+07
QHLS313	6868.9	7.5	3.8	1.67E+05	5.41E+03	2.99E+06	6.70E+08	1.90E+07
QHLS315	6919.9	7.1	5.5	5.28E+05	2.06E+05	9.60E+06	1.39E+08	4.84E+07
QHLS317	6971.3	6.4	3.5	4.91E+05	6.40E+04	9.09E+06	6.14E+08	4.62E+06
QHLS319	7023.1	6.5	4.1	1.77E+05	5.77E+04	3.35E+06	6.72E+07	2.39E+06
QHLS321	7075.3	7.1	3.3	3.32E+05	1.06E+05	6.38E+06	1.21E+08	2.27E+07
QHLS323	7127.8	6.3	3.3	3.88E+05	4.02E+05	7.61E+06	6.84E+08	3.39E+08
QHLS325	7180.7	6.5	5.9	3.44E+05	2.18E+04	6.87E+06	1.22E+08	3.64E+07
QHLS327	7234.1	6.8	4.7	6.70E+05	1.48E+05	1.36E+07	5.93E+08	2.94E+07
QHLS329	7287.8	nm	6.9	3.12E+05	1.05E+05	6.45E+06	5.88E+07	5.96E+06
QHLS331	7341.9	nm	4.5	2.17E+05	6.28E+04	4.57E+06	9.33E+08	3.99E+06
QHLS333	7396.5	nm	5.0	3.78E+05	3.24E+04	8.13E+06	2.97E+08	8.02E+07
QHLS335	7451.4	6.2	3.7	2.59E+05	2.59E+04	5.66E+06	6.30E+08	4.69E+06
QHLS337	7506.8	nm	4.9	5.60E+04	4.28E+04	1.25E+06	1.16E+08	4.85E+07
QHLS339	7562.5	5.8	4.5	7.97E+05	4.30E+04	1.81E+07	1.56E+08	2.80E+05
QHLS341	7618.7	8.6	3.8	5.56E+05	1.05E+05	1.29E+07	3.02E+08	7.83E+07
QHLS343	7675.3	7.3	2.3	3.57E+05	1.27E+05	8.40E+06	5.65E+08	4.47E+07
QHLS345	7732.3	7.6	3.8	6.60E+05	8.08E+04	1.58E+07	3.11E+08	1.47E+06
QHLS347	7789.7	7.0	3.6	1.78E+05	1.05E+05	4.35E+06	4.00E+08	4.71E+07

QHLS349	7847.6	6.9	3.1	1.12E+06	3.94E+05	2.78E+07	2.00E+08	1.92E+07
QHLS351	7905.9	6.4	4.1	no dna	no dna	no dna	no dna	no dna
QHLS353	7964.6	6.4	7.6	5.10E+05	2.40E+05	1.32E+07	2.89E+08	5.91E+07
QHLS355	8023.7	6.2	4.3	1.23E+05	4.10E+03	3.24E+06	7.25E+08	2.46E+07
QHLS357	8083.3	nm	6.2	1.35E+06	2.00E+05	3.62E+07	5.43E+08	5.35E+07
QHLS359	8143.4	5.8	4.3	5.35E+04	1.48E+04	1.46E+06	6.18E+08	6.77E+07
QHLS361	8203.9	4.6	6.2	3.31E+05	6.21E+04	9.20E+06	4.90E+08	2.15E+06
QHLS363	8264.8	nm	5.6	2.95E+05	9.16E+04	8.36E+06	4.49E+08	1.50E+08
QHLS365	8326.2	6.0	3.5	8.84E+04	9.35E+03	2.55E+06	6.76E+08	1.77E+07
QHLS367	8388.0	5.2	3.9	6.29E+04	5.10E+04	1.85E+06	2.42E+07	8.57E+06
QHLS369	8450.3	5.8	3.0	2.82E+04	6.06E+03	8.44E+05	2.95E+08	7.78E+06
QHLS371	8513.1	5.6	4.8	6.18E+04	9.38E+01	1.88E+06	2.01E+08	1.04E+07
QHLS373	8576.3	5.7	4.5	2.00E+05	3.74E+04	6.22E+06	2.12E+08	8.56E+07
QHLS375	8640.0	6.4	3.2	no dna	no dna	no dna	no dna	no dna
QHLS377	8704.2	6.2	4.5	1.11E+04	2.77E+03	3.56E+05	2.25E+07	3.18E+06
QHLS379	8768.8	5.9	2.8	no dna	no dna	no dna	no dna	no dna
QHLS381	8834.0	6.7	3.7	3.74E+05	3.43E+04	1.25E+07	4.79E+07	1.44E+07
QHLS383	8899.6	8.3	1.5	no dna	no dna	no dna	no dna	no dna
QHLS385	8965.7	9.6	3.0	2.08E+05	8.92E+04	7.22E+06	1.12E+08	1.12E+07
QHLS387	9032.3	9.6	nm	no dna	no dna	no dna	no dna	no dna
QHLS389	9099.4	8.3	2.5	3.23E+03	5.83E+02	1.16E+05	1.74E+07	5.42E+06
QHLS391	9166.9	7.1	4.5	1.40E+04	6.05E+03	5.13E+05	2.16E+07	9.57E+05
QHLS393	9235.0	6.0	2.2	no dna	no dna	no dna	no dna	no dna
QHLS395	9303.6	5.9	2.4	2.31E+04	7.14E+03	8.79E+05	1.47E+07	2.88E+06
QHLS397	9372.7	5.2	4.3	no dna	no dna	no dna	no dna	no dna
QHLS399	9442.3	5.6	2.2	7.08E+04	2.63E+04	2.79E+06	7.89E+06	3.31E+06
QHLS401	9512.5	6.2	nm	no dna	no dna	no dna	no dna	no dna
QHLS403	9583.1	6.6	2.5	7.33E+03	4.47E+02	3.00E+05	1.39E+08	1.50E+06
QHLS405	9654.3	5.6	2.8	5.89E+03	2.67E+03	2.46E+05	3.41E+06	1.26E+06
QHLS407	9726.0	6.1	2.7	7.22E+03	4.55E+03	3.07E+05	2.80E+07	3.90E+06

QHLS409	9798.2	5.9	2.4	5.94E+04	4.00E+04	2.57E+06	1.39E+08	5.01E+05
QHLS411	9871.0	5.5	2.7	1.98E+04	1.10E+04	8.74E+05	4.30E+06	6.32E+05
QHLS413	9944.3	5.3	2.1	2.07E+04	1.93E+03	9.30E+05	1.05E+08	6.67E+05
QHLS415	10018.2	5.5	3.9	1.33E+05	6.66E+04	6.06E+06	7.10E+06	1.21E+06
QHLS417	10092.6	5.3	4.4	5.81E+04	3.22E+03	2.70E+06	2.70E+07	2.67E+06
QHLS419	10167.6	5.1	3.4	1.28E+05	4.22E+04	6.06E+06	3.70E+08	1.13E+07
QHLS421	10243.1	5.3	3.2	5.14E+05	1.30E+05	2.48E+07	2.17E+08	5.60E+06
QHLS423	10319.2	5.1	2.9	1.90E+05	4.01E+04	9.34E+06	4.98E+08	3.50E+07
QHLS425	10395.8	5.5	3.1	7.88E+04	2.69E+04	3.95E+06	4.43E+07	7.12E+06
QHLS427	10473.0	5.5	3.0	5.56E+05	3.60E+04	2.84E+07	8.19E+07	1.11E+07
QHLS429	10550.8	5.5	3.7	5.75E+05	2.93E+04	2.99E+07	5.24E+08	9.82E+06
QHLS431	10629.2	nm	3.8	1.69E+05	6.56E+04	8.95E+06	5.57E+08	2.21E+06
QHLS433	10708.1	nm	2.1	4.67E+05	6.25E+03	2.52E+07	9.92E+07	2.25E+06
QHLS435	10787.7	9.0	1.8	no dna	no dna	no dna	no dna	no dna
QHLS439	10948.5	9.6	3.1	1.89E+06	2.76E+05	1.08E+08	1.59E+08	7.94E+07
QHLS443	11111.8	9.0	1.5	no dna	no dna	no dna	no dna	no dna
QHLS447	11277.4	10.5	nm	no dna	no dna	no dna	no dna	no dna
QHLS451	11445.6	nm	1.7	1.68E+06	3.90E+05	1.07E+08	1.21E+08	4.53E+07
QHLS453	11530.6	6.8	3.4	no dna	no dna	no dna	no dna	no dna
QHLS455	11616.2	5.9	1.9	4.15E+06	1.10E+06	2.74E+08	6.59E+07	8.59E+06
QHLS457	11702.5	6.1	1.3	no dna	no dna	no dna	no dna	no dna
QHLS459	11789.4	5.2	1.9	1.08E+06	7.12E+04	7.39E+07	1.21E+08	1.60E+07
QHLS461	11877.0	5.7	1.8	no dna	no dna	no dna	no dna	no dna
QHLS463	11965.2	4.9	1.9	6.11E+05	5.29E+04	4.35E+07	1.55E+08	8.31E+07
QHLS465	12054.1	5.1	0.7	no dna	no dna	no dna	no dna	no dna
QHLS467	12143.6	5.4	1.6	2.38E+06	4.10E+05	1.76E+08	2.37E+08	5.57E+07
QHLS469	12233.8	5.6	nm	no dna	no dna	no dna	no dna	no dna
QHLS471	12324.7	nm	1.9	8.00E+04	3.20E+04	6.12E+06	4.49E+08	8.53E+06
QHLS473	12416.2	4.7	1.8	3.90E+05	1.56E+04	3.04E+07	7.39E+07	3.28E+07
QHLS475	12508.4	4.7	1.6	3.80E+04	2.32E+02	3.02E+06	4.98E+08	4.95E+07

QHLS477	12601.3	4.7	1.9	5.63E+04	4.03E+04	4.55E+06	5.22E+08	8.28E+05
QHLS479	12694.9	4.2	nm	2.58E+05	8.02E+04	2.13E+07	4.34E+07	1.07E+06
QHLS481	12789.2	4.6	1.3	5.77E+04	1.34E+04	4.85E+06	3.50E+08	1.00E+07
QHLS483	12884.2	4.4	2.3	1.97E+05	6.44E+04	1.69E+07	3.45E+07	3.64E+06
QHLS485	12979.9	nm	nm	1.93E+05	8.97E+04	1.68E+07	5.82E+07	1.63E+07
QHLS487	13076.3	4.5	2.2	5.71E+04	1.84E+04	5.07E+06	3.95E+07	1.15E+06
QHLS489	13173.5	4.1	nm	no dna	no dna	no dna	no dna	no dna
QHLS491	13271.3	4.1	1.8	no dna	no dna	no dna	no dna	no dna
QHLS493	13369.9	4.3	nm	4.48E+04	1.79E+04	4.20E+06	4.12E+08	8.72E+06
QHLS495	13469.2	4.3	2.0	5.60E+04	8.77E+03	5.35E+06	4.80E+08	9.62E+06
QHLS497	13569.2	4.4	1.7	1.36E+05	9.31E+04	1.32E+07	4.47E+07	1.39E+07
QHLS499	13670.0	4.1	2.2	2.16E+05	7.09E+04	2.14E+07	3.22E+07	7.05E+06
QHLS501	13771.5	4.1	1.5	1.30E+05	4.02E+04	1.31E+07	5.32E+07	2.13E+05
QHLS503	13873.8	4.1	nm	4.24E+04	1.34E+04	4.36E+06	5.15E+08	1.16E+07
QHLS505	13976.9	nm	1.2	9.44E+04	9.03E+03	9.88E+06	3.47E+08	2.03E+07
QHLS507	14080.7	3.9	1.9	3.20E+05	7.13E+04	3.42E+07	4.66E+07	4.12E+06
QHLS509	14185.3	3.9	1.9	1.76E+05	4.89E+04	1.91E+07	4.29E+07	2.08E+06
QHLS511	14290.6	3.7	1.7	3.16E+04	8.32E+03	3.50E+06	3.09E+08	1.52E+07
QHLS513	14396.8	3.8	nm	9.39E+04	2.51E+04	1.06E+07	2.50E+08	1.22E+07
QHLS515	14503.7	3.7	nm	2.31E+04	1.16E+03	2.65E+06	1.91E+08	1.13E+07
QHLS517	14611.4	3.4	1.7	no dna	no dna	no dna	no dna	no dna
QHLS519	14720.0	3.2	2.1	7.31E+04	3.90E+04	8.70E+06	1.81E+07	7.94E+05
QHLS521	14829.3	3.6	1.8	7.31E+04	3.90E+04	8.87E+06	1.81E+07	7.94E+05
QHLS523	14939.4	3.3	1.8	6.33E+04	2.00E+04	7.82E+06	3.26E+07	1.23E+07
QHLS525	15050.4	3.3	1.8	1.25E+05	7.63E+04	1.57E+07	2.04E+07	2.61E+06
QHLS527	15162.2	3.5	1.0	1.35E+05	6.80E+03	1.73E+07	2.38E+08	2.14E+07
QHLS529	15274.8	3.2	1.9	5.08E+05	2.15E+05	6.63E+07	2.31E+07	2.47E+05
QHLS531	15388.2	2.6	1.8	4.57E+05	2.59E+05	6.08E+07	2.65E+07	7.39E+06
QHLS533	15502.5	3.2	nm	1.49E+05	6.71E+04	2.02E+07	3.91E+08	2.86E+07
QHLS535	15617.7	nm	1.8	1.06E+05	2.37E+04	1.46E+07	1.86E+08	3.69E+07

QHLS537	15733.7	3.1	1.7	7.68E+05	5.89E+04	1.08E+08	4.17E+07	9.83E+06
QHLS539	15850.5	3.1	1.0	1.66E+05	8.06E+03	2.37E+07	2.33E+08	7.54E+06
QHLS541	15968.3	3.5	1.5	1.62E+06	1.75E+05	2.36E+08	3.79E+07	1.46E+07
QHLS543	16086.9	3.5	1.7	no dna	no dna	no dna	no dna	no dna
QHLS545	16206.4	3.2	1.8	7.48E+05	9.75E+04	1.13E+08	1.69E+07	4.96E+06
QHLS547	16326.7	3.0	0.4	no dna	no dna	no dna	no dna	no dna
QHLS549	16448.0	3.2	1.7	1.53E+06	2.17E+05	2.40E+08	2.45E+07	2.05E+06
QHLS551	16570.2	3.1	0.1	no dna	no dna	no dna	no dna	no dna
QHLS553	16693.2	3.0	1.9	2.36E+06	1.96E+05	3.85E+08	1.87E+07	3.80E+06
QHLS555	16817.2	2.9	1.6	8.89E+05	2.43E+03	1.48E+08	1.36E+07	2.45E+06
QHLS557	16942.1	3.1	0.1	no dna	no dna	no dna	no dna	no dna
QHLS559	17068.0	2.9	2.0	4.65E+05	2.24E+05	8.01E+07	1.74E+07	7.04E+06
QHLS561	17194.7	3.0	0.3	no dna	no dna	no dna	no dna	no dna
QHLS563	17322.5	3.2	1.5	6.03E+05	1.86E+04	1.08E+08	1.37E+07	1.45E+06
QHLS565	17451.1	3.0	0.5	no dna	no dna	no dna	no dna	no dna
QHLS567	17580.7	2.9	1.7	2.39E+05	1.51E+04	4.42E+07	1.02E+08	2.25E+07
QHLS569	17711.3	3.2	0.9	4.11E+04	2.43E+04	7.77E+06	7.58E+08	3.33E+07
QHLS571	17842.9	3.6	1.3	9.47E+05	9.71E+04	1.82E+08	4.80E+07	6.84E+04
QHLS573	17975.4	nm	1.2	1.03E+06	2.56E+05	2.02E+08	4.54E+07	3.63E+05
QHLS575	18108.9	nm	nm	no dna	no dna	no dna	no dna	no dna
QHLS577	18243.4	8.1	1.4	3.78E+04	8.26E+03	7.69E+06	3.52E+07	4.02E+06
QHLS579	18378.9	nm	0.5	2.19E+04	1.70E+03	4.53E+06	3.34E+08	1.31E+07

Table S2 Ecological estimates of amoA gene libraries from the Qinghai Lake sediments

Libraries	Clones	Observed OTUs	Coverage	Simpson	Shannon	Evenness	Chao 1
QHLS007	22	4	95%	0.6	1.1	0.8	4.0
QHLS015	22	3	100%	0.6	1.0	0.9	3.0
QHLS029	21	3	95%	0.5	0.8	0.7	3.0
QHLS031	24	4	100%	0.5	0.9	0.6	4.0
QHLS043	21	3	100%	0.6	1.0	0.9	3.0
QHLS053	23	4	100%	0.6	1.2	0.8	4.0
QHLS063	24	4	100%	0.7	1.3	0.9	4.0
QHLS073	23	2	100%	0.5	0.6	1.0	2.0
QHLS087	23	3	100%	0.5	0.8	0.8	3.0
QHLS101	25	4	96%	0.6	1.0	0.7	4.0
QHLS115	23	5	87%	0.5	1.0	0.6	8.0
QHLS129	19	3	100%	0.5	0.9	0.8	3.0
QHLS147	22	4	91%	0.6	1.0	0.7	5.0
QHLS151	22	5	95%	0.6	1.2	0.7	5.0
QHLS157	22	5	91%	0.7	1.3	0.7	6.0
QHLS161	22	3	95%	0.5	0.8	0.8	3.0
QHLS169	24	3	100%	0.5	0.8	0.8	3.0
QHLS181	23	2	100%	0.2	0.3	0.7	2.0
QHLS187	22	3	100%	0.6	1.0	0.9	3.0
QHLS191	24	4	96%	0.5	1.0	0.7	4.0
QHLS203	22	3	100%	0.6	1.0	0.9	3.0
QHLS221	23	3	96%	0.5	0.8	0.7	3.0
QHLS223	22	2	100%	0.5	0.7	1.0	2.0
QHLS231	22	2	100%	0.5	0.7	1.0	2.0
QHLS237	24	2	100%	0.5	0.7	1.0	2.0
QHLS243	24	2	100%	0.3	0.5	0.8	2.0
QHLS257	20	1	100%	0.0	0.0	1.0	1.0
QHLS269	22	3	100%	0.7	1.1	1.0	3.0
QHLS283	22	3	100%	0.6	1.0	0.9	3.0
QHLS291	10	2	100%	0.5	0.7	1.0	2.0
QHLS299	14	2	100%	0.2	0.4	0.8	2.0
QHLS311	22	2	100%	0.4	0.6	0.9	2.0
QHLS319	17	2	100%	0.3	0.5	0.8	2.0
QHLS329	23	3	100%	0.4	0.7	0.7	3.0
QHLS339	21	2	100%	0.3	0.5	0.8	2.0
QHLS349	20	3	100%	0.5	0.9	0.8	3.0
QHLS363	24	3	96%	0.2	0.3	0.5	4.0
QHLS371	24	2	100%	0.2	0.3	0.7	2.0
QHLS377	16	1	100%	0.0	0.0	1.0	1.0
QHLS381	23	2	100%	0.5	0.7	1.0	2.0
QHLS389	24	1	100%	0.0	0.0	1.0	1.0
QHLS399	24	2	100%	0.5	0.7	1.0	2.0
QHLS415	23	2	96%	0.1	0.2	0.6	2.0
QHLS427	24	3	100%	0.6	0.9	0.8	3.0
QHLS439	22	2	100%	0.5	0.7	1.0	2.0
QHLS455	24	3	96%	0.5	0.8	0.7	3.0
QHLS463	23	4	91%	0.5	0.9	0.6	5.0
QHLS477	16	2	100%	0.3	0.5	0.8	2.0
QHLS491	21	1	100%	0.0	0.0	1.0	1.0
QHLS501	22	1	100%	0.0	0.0	1.0	1.0
QHLS525	24	1	100%	0.0	0.0	1.0	1.0
QHLS537	24	2	100%	0.4	0.6	0.9	2.0

QHLS553	24	2	100%	0.3	0.5	0.8	2.0
QHLS567	24	1	100%	0.0	0.0	1.0	1.0
QHLS573	24	2	100%	0.2	0.3	0.7	2.0
QHLS577	24	1	100%	0.0	0.0	1.0	1.0
